# From Ice to Perfusion: A Narrative Review of Modern Heart Preservation Systems

**DOI:** 10.3390/jcm15208114

**Published:** 2026-10-19

**Authors:** Raffaele Barbato, Benedetto Ferraresi, Mohamad Jawabra, Francesco Loreni, Alessandro Strumia, Marcello Bergonzini, Giuseppe Santarpino, Massimo Chello

**Affiliations:** 1 Unit of Cardiac Surgery, Department of Medicine, Fondazione Policlinico Universitario Campus Bio-Medico, 00128 Rome, Italy; benedetto.ferraresi@unicampus.it (B.F.); mohamad.jawabra@policlinicocampus.it (M.J.);; 2 Unit of Cardiac Surgery, AOPG Santa Maria della Misericordia Hospital, 06156 Perugia, Italy; francesco.loreni@ospedale.perugia.it (F.L.); marcello.bergonzini@ospedale.perugia.it (M.B.); 3 Department of Anesthesia and Intensive Care, Fondazione Policlinico Universitario Campus Bio-Medico, 00128 Rome, Italy; a.strumia@policlinicocampus.it; 4 Department of Experimental and Clinical Medicine, University “Magna Græcia” of Catanzaro, 88100 Catanzaro, Italy; santarpino@unicz.it

**Keywords:** heart transplantation, heart preservation, cardiac surgery, cardioplegic solutions, normothermic heart perfusion

## Abstract

Donor heart preservation is still a critical determinant of outcomes after heart transplantation, particularly as transplant programs increasingly rely on extended-criteria and donation-after-circulatory-death donors. This narrative review provides an overview of the evolution of donor heart preservation, from conventional static cold storage to contemporary technologies designed to reduce ischemia–reperfusion injury, improve graft assessment, and expand organ utilization. We discuss the physiological rationale and clinical evidence supporting commonly used cardioplegic solutions, including University of Wisconsin, Celsior, and histidine–tryptophan–ketoglutarate solutions. We then examine advanced preservation strategies, including controlled hypothermic preservation systems and ex vivo normothermic machine perfusion, which allow improved temperature control, continuous monitoring, functional assessment, and, in the case of machine perfusion, potential metabolic optimization of the graft during transport. Current evidence suggests that these technologies could extend acceptable preservation times, facilitate long-distance procurement, and support the use of marginal and DCD grafts. However, their widespread adoption is limited by logistical complexity, costs, and the need for further independent studies evaluating long-term clinical outcomes. Modern preservation platforms can represent a major paradigm shift in heart transplantation, with the potential to improve graft availability and post-transplant outcomes.

## 1. Introduction

Heart transplantation remains the definitive treatment for patients with end-stage heart failure, achieving annual survival rates above 85% and 10-year survival approaching 70% [1]. The success of transplantation critically depends on graft quality at implantation, which in turn is influenced by preservation techniques and ischemic duration. (Figure 1). In recent years, the growing demand for organs necessitated the inclusion of extended-criteria and donation-after-circulatory-death (DCD) donors: in this setting, optimal donor heart preservation remains a major challenge [2,3].

For more than five decades, static cold storage (SCS) using cardioplegic preservation solutions such as University of Wisconsin (UW), Celsior, and histidine–tryptophan–ketoglutarate (HTK) has remained the gold standard for cardiac allograft transport [4]. This technique, based on immersion of the donor heart in a crystalloid solution on ice at approximately 4 °C, slows metabolic activity to about 10% of baseline levels but doesn’t completely arrest it. When cold ischemic times exceed 4–6 h, the risk of primary graft dysfunction (PGD) increases markedly, leading to higher rates of early mortality [1,4]. Furthermore, temperature fluctuations and direct contact with ice may cause freezing injury, cell swelling, and protein denaturation—all contributing to post-transplant graft dysfunction [1]. To overcome the intrinsic limitations of conventional SCS, new controlled and dynamic preservation systems have been developed. The Paragonix SherpaPak Cardiac Transport System (SCTS) represents an evolution of static preservation, maintaining a strictly controlled temperature range between 4 °C and 8 °C and eliminating temperature gradients within the myocardial tissue. Multicentre clinical data have shown that SherpaPak preservation is associated with reduced myocardial injury, lower rates of bradyarrhythmia and moderate-to-severe PGD, and improved endothelial integrity, even in marginal or high-risk grafts with prolonged ischaemic times [5,6,7]. Furthermore, its use in DCD heart transplantation has demonstrated favourable functional outcomes after transport durations of up to 3.5 h [3]. In parallel, the advent of ex vivo normothermic machine perfusion using the TransMedics Organ Care System (OCS) has ushered in a new era of dynamic donor heart preservation. The OCS maintains the heart in a beating, perfused state with oxygenated blood during transport, thereby reducing prolonged cold ischemic exposure and allowing real-time assessment of graft function [8,9]. Clinical experience has demonstrated that the OCS significantly reduces cold ischemic time and improves outcomes in high-risk recipients, particularly in patients bridged to transplant with mechanical circulatory support, but it requires more complex logistics and higher costs [8]. Recent research into hypothermic and normothermic perfusion strategies suggests that combining continuous perfusion with pharmacologic interventions may further enhance myocardial viability, microvascular integrity, and expand the use of marginal donor hearts [1,10]. This narrative review aims to critically compare the main heart preservation strategies—traditional ice-cold static storage, Paragonix SherpaPak controlled hypothermia, and the TransMedics Organ Care System—highlighting their physiological principles, clinical outcomes, advantages, limitations, and future perspectives in the improvement of graft preservation and expansion of the donor heart pool.

## 2. Materials and Methods

This narrative review is based on a structured literature search conducted across PubMed/MEDLINE, Scopus, and Web of Science to identify relevant studies on donor heart preservation strategies. The search focused on articles addressing static cold storage, cardioplegic preservation solutions, controlled hypothermic transport systems, and ex vivo machine perfusion platforms in heart transplantation. Search terms included combinations of *heart transplantation*, *donor heart preservation*, *static cold storage*, *University of Wisconsin solution*, *Celsior*, *HTK*, *SherpaPak*, *controlled hypothermia*, *Organ Care System*, *normothermic perfusion*, *hypothermic oxygenated perfusion*, *XVIVO*, and *donation after circulatory death*. Priority was given to original clinical studies, multicentre registries, randomized trials, and relevant translational studies published in English. Additional articles were identified through manual screening of the reference lists of selected papers. Articles were selected on the basis of their relevance to the scope of the review, with particular attention to preservation mechanisms, technical characteristics, and post-transplant clinical outcomes. The images included in this article were generated with the assistance of ChatGPT (Pro version 28.8). The authors carefully reviewed, edited, and supervised all AI-generated outputs and take full responsibility for the accuracy, integrity, and final content of the image.

## 3. Cardioplegic Preservation Solutions in Heart Transplantation: Physiological Principles, Clinical Evidence and Practical Considerations

### 3.1. Physiological Rationale and Mechanisms of Protection

Static cold storage (SCS) represents the global standard of care due to its simplicity, cost-effectiveness and reliability. The physiological goal of SCS is to rapidly induce diastolic arrest, decrease myocardial temperature and suppress metabolic activity, thereby limiting ischemia–reperfusion injury [6]. Preservation solutions primarily differ in their ionic composition and buffering capacity. They are commonly classified as either intracellular-type (high K^+^, low Na^+^), as in the University of Wisconsin (UW) solution and the histidine–tryptophan–ketoglutarate (HTK) solution, or extracellular-type (plasma-like electrolyte composition), as in the Celsior solution [4]. These solutions contain various combinations of impermeable agents, antioxidants, osmotic agents, and buffering systems, specifically designed to stabilise cell membranes, prevent oedema, and scavenge free radicals. Cold storage dramatically reduces myocardial oxygen demand by approximately 90% per 10 °C drop, but it does not completely block metabolism. Beyond 4–6 h of cold ischaemic time (CIT), the cumulative oxygen deficit and acidosis lead to mitochondrial dysfunction and primary graft dysfunction (PGD) [11]. Therefore, the choice of preservation solution and thermal management both critically influence post-transplant graft recovery.

### 3.2. Comparative Clinical Evidence

Over the past two decades, multiple clinical studies and meta-analyses have compared the performance of UW, Celsior, and HTK solutions in heart transplantation. The University of Wisconsin (UW) solution has been shown to consistently deliver superior outcomes. In the systematic review and meta-analysis by Li et al., which included 5301 grafts for the UW-versus-Celsior comparison, UW was associated with significantly better 30-day survival (HR 1.16, 95% CI 1.11–1.22; *p* < 0.00001) and 90-day survival (risk difference 0.03, 95% CI 0.01–0.05; *p* = 0.002), as well as less ischemic necrosis on early post-transplant biopsy (RD −0.07, 95% CI −0.08 to −0.05; *p* < 0.00001). In recipients of suboptimal grafts, UW also showed higher survival than Celsior at 30 days (94.31% vs. 91.26%; *p* < 0.005) and at 1 year (84.19% vs. 80.19%; *p* < 0.01). Compared with HTK, UW was likewise associated with better short- and long-term outcomes: in one study, 30-day and 6-month survival were 90% and 88% with UW versus 81% and 78% with HTK (*p* < 0.05), while registry-based data showed 1-, 5-, 10-, and 15-year survival of 80.1%, 72.3%, 58.5%, and 46.4% with UW versus 66.1%, 58.7%, 47.3%, and 36.4% with HTK (*p* < 0.001) [4,6,12]. In contrast, HTK and Celsior have shown comparable performance in terms of early graft dysfunction and in-hospital mortality [4]. However, histopathological analyses indicate greater endothelial activation and inflammatory gene expression in hearts preserved with Celsior than with UW; these findings correlate with higher postoperative vasopressor requirements [6]. Taken together, these data suggest that the impermeables and antioxidants contained in UW provide superior protection against ischaemic and reperfusion injury (Figure 2).

### 3.3. Advantages and Limitations of the Main Solutions

#### 3.3.1. University of Wisconsin (UW)

Advantages:

Designed as an intracellular-type solution, UW remains the reference standard for static cold storage. Its formulation containing lactobionate, raffinose, allopurinol and glutathione provides powerful antioxidant and osmotic protection. Clinical and histological evidence consistently demonstrates superior graft survival and reduced ischaemic necrosis compared to Celsior and HTK [4].

Limitations:

The relatively high viscosity of UW can delay the initial distribution of the solution and the achievement of cardioplegic arrest when UW is used alone. To overcome this limitation, Wang et al. proposed a crystalloid cardioplegia plus University of Wisconsin solution approach (CC + UW), consisting of an initial cardioplegic flush with crystalloid cardioplegia followed by static cold storage in UW solution [13]. In their experimental model, this strategy induced cardiac arrest within seconds, whereas the use of UW alone required a longer time for contraction to cease. As with all static cold storage techniques, however, its protective effect still declines as cold ischemic time is prolonged [11].

#### 3.3.2. Celsior

Advantages:

Designed as an extracellular-type solution, Celsior combines antioxidants such as glutathione and mannitol with buffering agents to maintain ionic balance. In the systematic review by Li et al., Celsior showed outcomes comparable to HTK with respect to early graft dysfunction and in-hospital mortality [5]. Specifically, pooled analysis of 147 hearts from two studies showed no significant difference in donor heart dysfunction between Celsior and HTK (risk difference −0.10, 95% CI −0.26 to 0.05; *p* = 0.20) and no significant difference in in-hospital mortality (risk difference −0.07, 95% CI −0.17 to 0.03; *p* = 0.19), although both outcomes tended to occur less frequently with Celsior. In addition, Cannata et al. reported no significant differences between Celsior and HTK in the need for intraoperative intra-aortic balloon pump or veno-arterial extracorporeal membrane oxygenation. Its low viscosity also favors rapid and uniform perfusion [4].

Limitations:

Compared with UW, Celsior has been associated with less favorable graft protection in some clinical studies. In the systematic review by Li et al., donor hearts preserved with UW showed less ischemic necrosis on early post-transplant biopsy than those preserved with Celsior [5]. Moreover, Wildhirt et al. reported higher endothelin (ET) and inducible nitric oxide synthase (iNOS) expression in grafts preserved with Celsior, together with a significantly greater requirement for vasoactive drugs to maintain stable post-transplant hemodynamics [14]. Kur et al. also reported greater myocardial damage with prolonged ischemic times (>300 min), supporting the view that UW may provide more effective metabolic protection during extended ischemia [6].

#### 3.3.3. Histidine–Tryptophan–Ketoglutarate (HTK)—Custodiol

Advantages.

Designed as an extracellular-type cardioplegia, histidine–tryptophan–ketoglutarate (HTK, Custodiol) is valued for its low viscosity, cost efficiency, and ease of administration, allowing a single-dose protocol that facilitates multiorgan procurement. In the systematic review by Li et al., HTK showed outcomes broadly comparable to Celsior with respect to early graft dysfunction and in-hospital mortality [5]. Specifically, pooled analysis of 147 hearts from two studies found no significant difference in donor heart dysfunction between HTK and Celsior (risk difference −0.10, 95% CI −0.26 to 0.05; *p* = 0.20) and no significant difference in in-hospital mortality (risk difference −0.07, 95% CI −0.17 to 0.03; *p* = 0.19), although both outcomes tended to occur less frequently with Celsior. In addition, Cannata et al. reported no significant differences between the two solutions in the need for intraoperative intra-aortic balloon pump or veno-arterial extracorporeal membrane oxygenation [4].

Limitations.

However, UW consistently outperforms HTK in both short- and long-term survival analyses. HTK’s limited buffering capacity and absence of impermeants may restrict its efficacy during prolonged ischemia, especially in marginal donor settings or when CIT exceeds 4–6 h.

### 3.4. Practical Implications for Clinical Use

In routine transplantation with conventional ischaemic times, UW remains the preferred solution due to its robust evidence base and superior histological protection. For institutions prioritising low viscosity and rapid flushing, HTK or Celsior remain valid alternatives, provided their limitations are acknowledged. In extended criteria donor (ECD) scenarios, or when prolonged transport times are anticipated, integrating an initial cardioplegic flush (CC + UW) and controlled hypothermic storage may minimise endothelial stress and PGD risk [10,11]. Future studies should focus on large, multicentre trials comparing modernised SCS protocols, including CC + UW, Celsior and HTK, under standardised, controlled-temperature conditions and evaluating their performance against dynamic preservation technologies, such as normothermic perfusion [6].

To sum up, cardioplegic preservation solutions remain central to myocardial protection during static cold storage for heart transplantation. Of the available options, the University of Wisconsin solution offers the most consistent clinical and histological benefits, providing improved early survival and reduced ischaemic injury compared to Celsior and HTK [4]. Emerging strategies, particularly CC + UW and controlled hypothermia, enhance protection by addressing physiological mechanisms beyond the chemical composition of the cardioplegic solution. Thus, a contemporary preservation strategy should integrate an optimal cardioplegic solution, an effective arrest technique, and stable thermal control to ensure maximal myocardial protection, even in the context of prolonged ischemia and marginal donors (Table 1).

## 4. Current Strategies for Organ Preservation and Transport

### 4.1. Paragonix SherpaPak Cardiac Transport System (SCTS)

The Surgical Cardiac Transport System (SCTS) (Figure 2) is a controlled hypothermia device for cardiac transport in which the graft is immersed and suspended in preservation solution within a sterile rigid container. Cooling is provided by phase-change materials that maintain a stable temperature between 4 and 8 °C, thereby avoiding direct contact with ice, local thermal gradients, and uncontrolled freezing injury. In the single-center study by Bitargil et al., the SherpaPak system maintained a mean transport temperature of 5.6 ± 0.8 °C and was associated with a significantly shorter ICU stay despite longer ischemic and cardiopulmonary bypass times (6.3 ± 5.3 vs. 7.5 ± 5.0 days, *p* = 0.01; ischemic time 230.1 ± 41 vs. 213.4 ± 35 min, *p* = 0.05). Although overall PGD rates were similar (26% vs. 32%, *p* = 0.4), no cases of severe PGD or mortality were observed in the SherpaPak group, whereas severe PGD occurred in 4% of patients preserved with conventional ice storage. In the multicenter GUARDIAN-Heart registry analysis by Shudo et al. (n = 569), SCTS preservation was associated with significantly lower severe PGD compared with ice cold storage (5.4% vs. 10.2%, *p* = 0.03), and in the propensity-matched cohort severe PGD remained significantly reduced (4.0% vs. 12.0%, *p* = 0.011), with a trend toward higher 1-year survival (94.0% vs. 88.7%, *p* = 0.10). In grafts exposed to ischemic times >4 h, SCTS was further associated with lower post-transplant MCS use (13.4% vs. 32.0%, *p* = 0.01), lower severe PGD (3.7% vs. 18.0%, *p* = 0.005), and better 30-day survival (100% vs. 94%, *p* = 0.02) [15]. Consistently, Lechiancole et al. reported in a high-risk donor–recipient cohort that controlled hypothermia with SherpaPak achieved similar 30-day mortality to conventional ice storage (3% vs. 3%, *p* = 0.99), but showed a trend toward lower moderate-to-severe graft dysfunction (7% vs. 20%, *p* = 0.08) and fewer bradyarrhythmias persisting >48 h (3% vs. 15%, *p* = 0.09), while severe cellular and endothelial injury on histology was absent in SherpaPak-preserved grafts [5,15].

#### 4.1.1. Advantages

The main advantages of SCTS are supported by both technical and clinical data. First, it maintains the graft at a controlled temperature between 4 and 8 °C, thereby avoiding direct contact with ice and the thermal fluctuations associated with conventional ice storage. In the GUARDIAN-Heart analysis by Shudo et al. [15], the mean preservation temperature with SCTS was 5.2 °C, while no equivalent temperature monitoring was available in the ice-cold storage group because this is not routinely measured in clinical practice. Second, the system provides continuous monitoring and traceability of the cold chain through its integrated digital platform. Third, favorable clinical signals have emerged in both registry-based and comparative analyses. In the overall GUARDIAN-Heart cohort, SCTS was associated with a significantly lower rate of severe PGD than ice cold storage (5.4% vs. 10.2%, *p* = 0.03), despite longer ischemic times (217 ± 48 vs. 193 ± 58 min, *p* < 0.001); in the propensity-matched cohort, severe PGD remained significantly reduced (4.0% vs. 12.0%, *p* = 0.011), with a lower overall post-transplant MCS use (18.7% vs. 26.7%, *p* = 0.098) and a trend toward better 1-year survival (94.0% vs. 88.7%, *p* = 0.10). In multivariable analysis, conventional ice storage was identified as an independent predictor of severe PGD, with a 3.4-fold higher risk compared with SCTS (OR 3.384, 95% CI 1.28–8.96; *p* = 0.014). These benefits were even more evident when ischemic time exceeded 4 h: SCTS was associated with lower post-transplant MCS use, lower severe PGD, and improved 30-day survival. Finally, comparative registry data suggest that SCTS may safely expand procurement distances and ischemic times without compromising short-term outcomes. In the study by Farhoud et al., hearts preserved with SCTS despite markedly longer ischemic times than conventional ice storage (4.6 vs. 2.3 h, *p* < 0.001) and much greater procurement distance (765 vs. 82 miles, *p* < 0.001) showed similar rates of PGD (18.2% vs. 20.8%, *p* = 0.58), post-transplant MCS use (20.3% vs. 25.1%, *p* = 0.34), hospital length of stay (24.8 vs. 24.7 days, *p* = 0.98), and 30-day survival (98.6% vs. 100%, *p* = 0.20), together with lower 24-h inotrope scores (13.6 vs. 17.6, *p* = 0.007) and less moderate/severe right ventricular dysfunction (15.7% vs. 31.1%, *p* = 0.002) [15,16].

#### 4.1.2. Disadvantages

The use of SCTS presents several disadvantages:

1. As a static preservation method, SCTS does not permit functional assessment or ‘resuscitation’ of the organ during transport.
2. There is a finite ischemic window: despite greater thermal safety, the intrinsic limitations of cold ischemia are not eliminated, and data beyond typical durations are still scarce.
3. Costs and logistics are higher than those of ice (due to the need for devices and training), and have been little explored in studies [15,16].
4. The evidence is predominantly observational and partly manufacturer-funded (e.g., the GUARDIAN registry), making it susceptible to bias; there is a lack of independent RCTs.

In summary, the SherpaPak system provides cold storage in a controlled manner (4–8 °C, without ice and with monitoring), offering plausible biological advantages and encouraging real-world clinical findings, despite limitations relating to the level of evidence and the static nature of the method [15].

### 4.2. TransMedics Organ Care System (OCS)

The Organ Care System (OCS) (Figure 3) is a pulsatile normothermic ex vivo perfusion platform designed to maintain the donor heart in near-physiological beating conditions during transport. After donor heart acceptance, the graft is first assessed in situ by echocardiography and hemodynamic evaluation. Before aortic cross-clamping, the donor is heparinized (300 IU/kg), the right atrial appendage is cannulated, and approximately 1.5 L of donor blood is collected to prime the OCS circuit; an additional 500–750 mL of warm blood is collected for the initial cardioplegic dose. Thus, the heart is initially arrested in the donor in the conventional way with cardioplegia, then explanted, cannulated at the aorta and pulmonary artery, and mounted on the OCS chamber. Once connected to the system, oxygenated donor blood is pumped into the aorta to perfuse the coronary arteries, while venous effluent returns through the coronary sinus, passes through the right atrium and right ventricle, and is collected from the pulmonary artery to complete the circuit. The graft is then reanimated to sinus rhythm and transported under continuous monitoring, with mean aortic pressure maintained at 60–90 mmHg and coronary flow at 650–850 mL/min. Arterial and venous lactate and blood gas samples are obtained approximately every 20–30 min to guide graft assessment during transport. On arrival at the recipient center, the heart is re-arrested with about 1 L of normothermic blood cardioplegia before implantation. In the series reported by Kaliyev et al., OCS preservation reduced total ischemic time from 210 ± 23 min with cold storage to 74.6 ± 13 min (*p* < 0.001), despite a significantly longer total out-of-body time (423 ± 67 vs. 210 ± 23 min, *p* = 0.002), supporting the rationale that OCS limits ischemia–reperfusion injury by replacing prolonged cold ischemia with continuous warm perfusion [12]. More broadly, temperature should be regarded as a central design variable in ex situ heart perfusion rather than simply a device setting. As summarized by Georghiou et al., normothermic machine perfusion at approximately 34 °C maintains the graft in a beating, near-physiological state and enables continuous metabolic and functional assessment, but this benefit is accompanied by higher myocardial oxygen and energy requirements, greater logistical complexity, and vulnerability to warm ischemic injury if perfusion is interrupted. By contrast, hypothermic oxygenated perfusion at approximately 8–10 °C markedly suppresses myocardial metabolic demand while maintaining oxygen delivery, which may limit ischemia–reperfusion injury and support preservation of cellular energy stores; however, real-time functional assessment is limited and prolonged hypothermia may be associated with reversible edema or cold-related mitochondrial stress. Subnormothermic perfusion, typically around 21 °C, has been proposed as an intermediate strategy that seeks to combine metabolic suppression with more physiological oxygenation and the potential for graft assessment, although clinical evidence and standardized protocols remain limited. Accordingly, the optimal temperature is unlikely to be universal and should be considered in relation to donor risk, expected preservation time, myocardial metabolic demand, ischemia–reperfusion injury, and the relative need for metabolic protection versus functional evaluation [2].

#### 4.2.1. Advantages

This system minimises cold ischemia and enables pre-implantation functional assessment using biomarkers and haemodynamic signals. In the study by Kaliyev et al., OCS reduced total ischaemic time from 210 ± 23 min to 74.6 ± 13 min (*p* < 0.001), despite a significantly longer total out-of-body time (423 ± 67 vs. 210 ± 23 min, *p* = 0.002). OCS use was also associated with a lower need for post-transplant mechanical circulatory support (24% vs. 60%, *p* = 0.02), while 30-day survival remained comparable between groups (96% vs. 100%, *p* = 0.5) [17].

#### 4.2.2. Disadvantages

Despite these potential advantages, OCS is substantially more resource-intensive than static cold storage. Its use requires dedicated equipment, trained personnel, blood-based circuit preparation, continuous monitoring, and more complex procurement and transport logistics. These requirements may limit availability, particularly in lower-volume centres or healthcare systems in which the additional cost cannot be readily justified. Cost-effectiveness therefore remains an important consideration, particularly when OCS is used for standard-risk donor hearts that could otherwise be safely transported using conventional preservation. In addition, although contemporary comparative and registry studies provide encouraging evidence, part of the available clinical literature derives from selected populations and sponsored post-approval registries. The relative contribution of the preservation platform itself, as opposed to donor–recipient selection and centre expertise, therefore remains difficult to isolate. Further independent comparative studies, long-term outcome analyses, and formal cost-effectiveness evaluations are needed to define which donor–recipient scenarios derive the greatest clinical benefit from OCS [17].

### 4.3. XVIVO Heart Preservation System

The XVIVO heart preservation platform (Figure 4) is based on hypothermic oxygenated perfusion (HOPE), a strategy designed to preserve donor hearts in a non-beating, metabolically suppressed state while continuously providing oxygenated perfusate. Unlike static cold storage, this approach aims to reduce ongoing ischaemic injury during transport while avoiding the logistical complexity of normothermic beating-heart perfusion. The strongest clinical evidence currently comes from the multicentre randomized NIHP2019 trial, in which HOPE was associated with lower rates of primary graft dysfunction, severe primary graft dysfunction, and major adverse cardiac transplant events within 30 days compared with conventional static cold storage [18]. In parallel, the Australian and New Zealand multicentre experience suggested that the XVIVO Heart Preservation System may safely support prolonged preservation times of 6 to 8 h, with acceptable early survival and graft function, although this was a non-randomized and single-arm study [19]. Preclinical and early translational studies have further supported the biological rationale for HOPE, including improved post-transplant ventricular performance and lower biochemical evidence of myocardial injury in donation-after-circulatory-death settings. Overall, the XVIVO system represents a promising addition to contemporary donor heart preservation, particularly for prolonged transport and potentially more complex donor scenarios; however, the available evidence remains heterogeneous, and some studies involve industry collaboration or early feasibility settings, so broader independent validation and longer-term outcome data are still needed before definitive conclusions can be drawn.

## 5. Discussion

The most recent data suggest that heart preservation for transplantation should no longer be viewed solely as a logistical step aimed at maintaining organ viability during transport. Rather, it is increasingly emerging as a therapeutic strategy that may influence organ transplantability, donor selection, and early post-transplant risk [1,19]. However, the current evidence base remains heterogeneous, with substantial differences in study design, donor and recipient selection, preservation protocols, and outcome definitions. In addition, a considerable proportion of the available studies on newer preservation and transport technologies derives from observational cohorts, registry analyses, or industry-sponsored investigations; this setting requires cautious interpretation of the reported benefits [15,16,17]. All evaluated studies are reported in Table 2. Three main considerations arise from the current literature.

1. Safe extension of ischaemic time and potential geographic expansion of procurement.

There is growing evidence that newer preservation strategies may help mitigate some of the risks traditionally associated with prolonged cold ischemia [11,15,16,18,19]. This appears to be driven, first, by improvements in the initial arrest and preservation phase. In the recent study by Wang et al., the combination of crystalloid cardioplegia and University of Wisconsin solution was associated with improved tolerance to prolonged cold ischemia, both experimentally and in clinical analyses [11]. Likewise, registry-based studies of controlled transport systems, particularly SherpaPak, have reported lower rates of severe primary graft dysfunction despite longer transport distances and ischaemic times [15,16]. However, these findings should be interpreted with caution, as they largely derive from non-randomized studies with potential selection bias, residual confounding, and heterogeneous preservation contexts. Therefore, while modern preservation strategies appear promising in extending acceptable procurement distances and ischaemic times, the extent to which they truly redefine conventional ischaemic thresholds remains to be established in more rigorous comparative studies.

2. Broader use of marginal and DCD donors.

The increasing use of preservation strategies in grafts that would previously have been considered suboptimal, including marginal donors and donors after circulatory death, is an important clinical improvement [3,17,18,19]. Emerging clinical experience suggests that these technologies may facilitate the safe utilization of such organs, especially when combined with structured reperfusion protocols and careful donor–recipient selection. Nevertheless, the supporting evidence remains heterogeneous, and results from highly selected cohorts may not be fully generalizable across centers with different expertise, logistics, and retrieval practices. As a result, although these approaches are contributing to donor pool expansion, they should still be regarded as evolving strategies rather than universally validated standards.

3. Organ protection may translate into clinically meaningful outcomes, but the strength of evidence varies.

More recent studies have begun to suggest that improved preservation may be associated not only with favorable physiological or biomarker profiles, but also with clinically relevant outcomes. The strongest evidence currently comes from the randomized multicentre NIHP2019 trial, in which hypothermic oxygenated perfusion reduced primary graft dysfunction and severe primary graft dysfunction compared with static cold storage [18]. In contrast, much of the evidence supporting controlled hypothermic transport systems and normothermic perfusion platforms comes from registries and observational comparisons. Although these studies consistently report encouraging trends in severe PGD, post-transplant mechanical circulatory support, and short-term survival, their interpretation is limited by heterogeneity in study populations, preservation strategies, and sponsorship patterns. Accordingly, these findings should be considered promising. In the future, further independent prospective studies are needed to clarify which preservation strategy is most beneficial for which donor–recipient scenario.

## 6. Cost-Effectiveness and Health-System Implications

The clinical adoption of advanced donor-heart preservation technologies should also be considered from a health-system and cost-effectiveness perspective. Compared with conventional static cold storage, controlled hypothermic transport and machine-perfusion platforms require additional expenditures related to the preservation device, disposable components, transport, trained personnel, staff education, and more complex procurement and operating-room logistics. These direct costs are particularly relevant for normothermic machine perfusion, which also requires dedicated technical expertise, blood-based circuit preparation, and continuous monitoring during transport. However, the economic impact of these technologies cannot be assessed solely on the basis of their acquisition and procedural costs. Potential downstream benefits may include increased utilization of donor hearts that might otherwise be declined, expansion of the effective donor pool, reduced preservation-related graft injury, lower rates of primary graft dysfunction, reduced need for post-transplant mechanical circulatory support, and potentially shorter intensive-care or hospital stays. If confirmed, these benefits could partially offset the additional costs associated with advanced preservation systems. At present, however, formal comparative cost-effectiveness data remain limited, and the magnitude of any economic benefit is likely to depend on donor and recipient risk, transport distance, institutional volume, local procurement organization, and the preservation strategy being compared. Consequently, advanced preservation technologies should not be assumed to be cost-effective solely because they improve selected preservation or short-term clinical outcomes. Future studies should incorporate formal health-economic analyses, including device and disposable costs, personnel and transport requirements, operating-room resource utilization, organ acceptance rates, primary graft dysfunction, mechanical circulatory support, intensive-care and hospital length of stay, and longer-term post-transplant outcomes. Defining the donor–recipient scenarios in which the incremental clinical benefit justifies the additional financial and logistical burden represents an important research priority.

## 7. Conclusions

The choice of heart preservation system in heart transplantation has evolved from being an only logistical issue to a complex decision based on selective and therapeutic considerations. The most modern strategies serve not only to ‘keep the organ alive until implantation’, but also to safely extend harvest times and distances, reliably use marginal and donor hearts after circulatory arrest, reduce primary graft dysfunction in the most critically ill recipients, and potentially provide a platform for metabolic optimization or targeted biological interventions before transplantation. The next step is to define how these technologies can be integrated sustainably and rationally into clinical practice, determine which donor–recipient scenarios derive the greatest benefit from each preservation strategy, and clarify their cost-effectiveness and long-term impact on graft function and recipient survival. By potentially increasing donor-heart utilization and facilitating the use of selected marginal and DCD grafts, modern preservation strategies may contribute to expansion of the effective donor pool. However, whether this translates into reductions in waiting-list mortality or overall transplant-related mortality remains to be demonstrated in prospective studies [6,7,11,15,17,18].

## Figures and Tables

**Figure 1:**
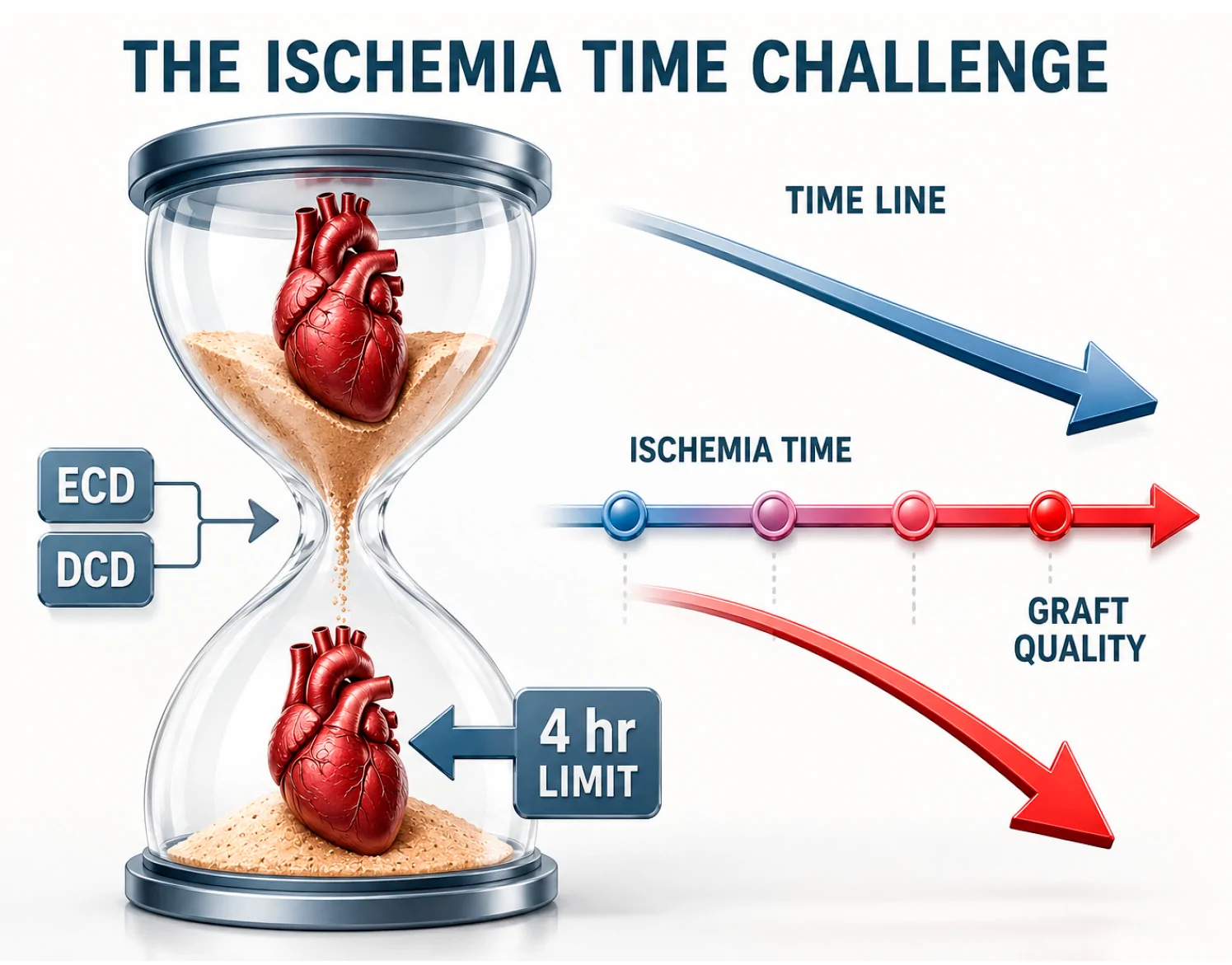
The ischemia time challenge in donor heart preservation.

**Figure 2:**
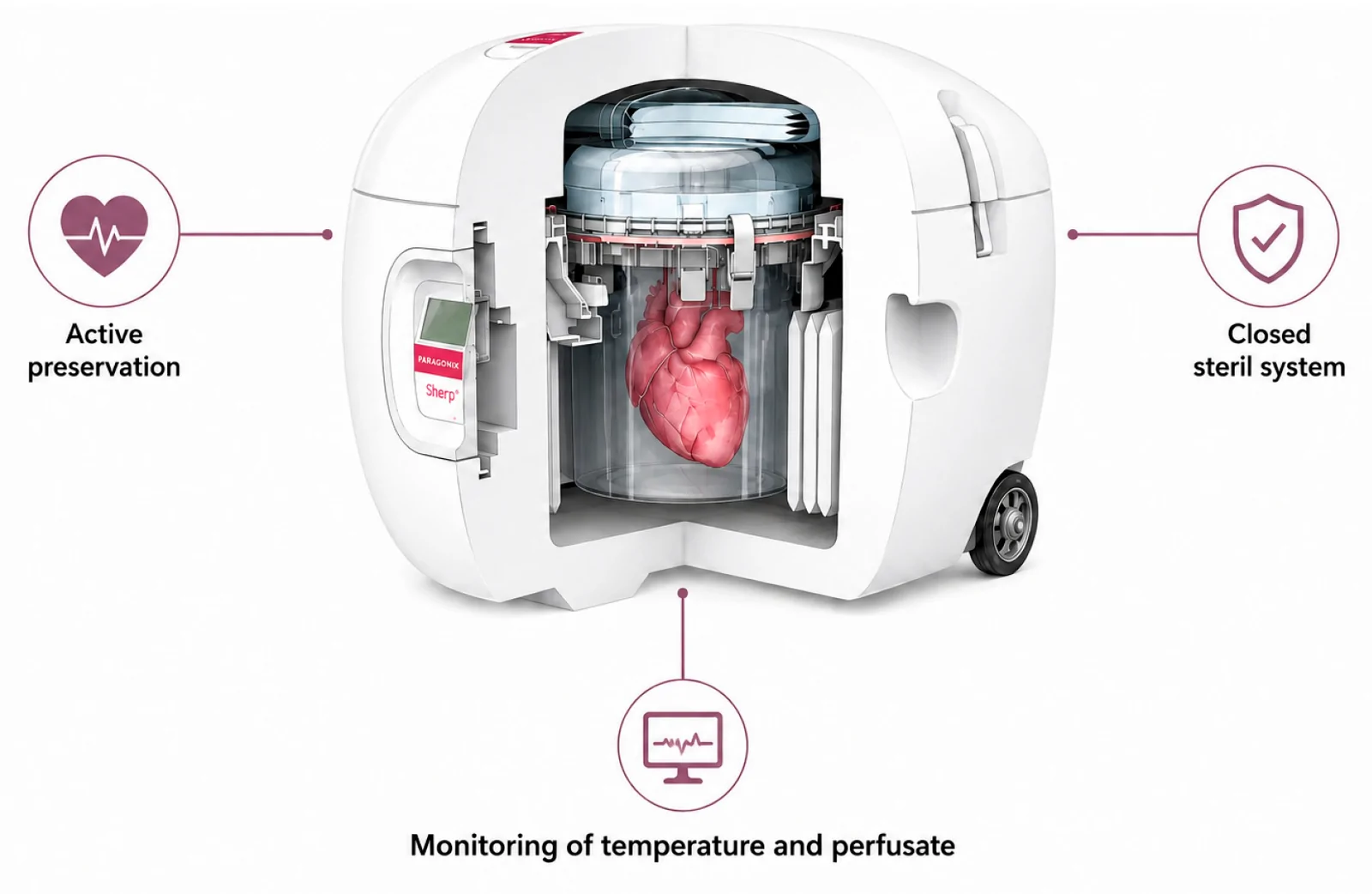
The Paragonix SherpaPak Cardiac Transport System.

**Figure 3:**
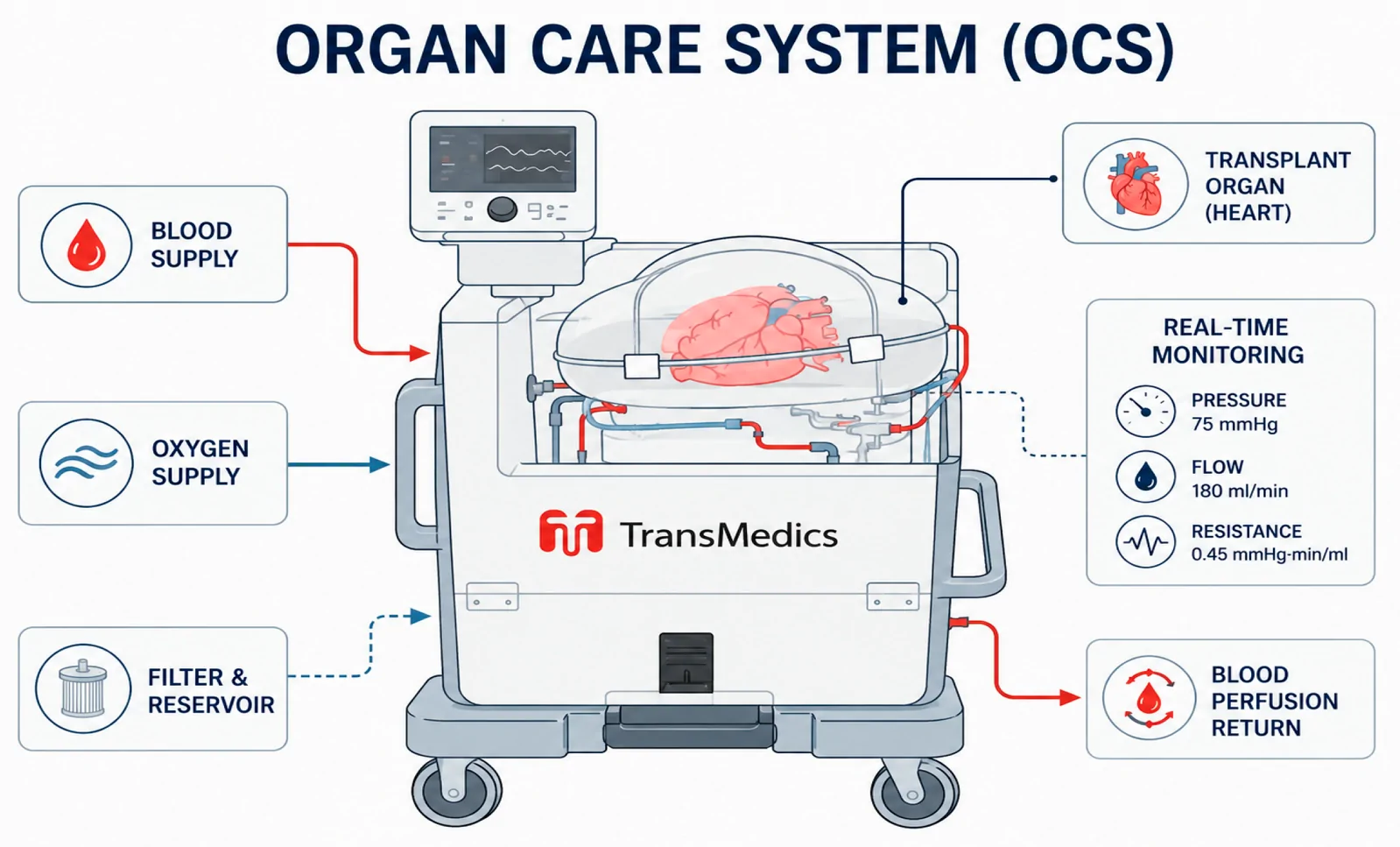
TransMedics Organ Care System (OCS).

**Figure 4:**
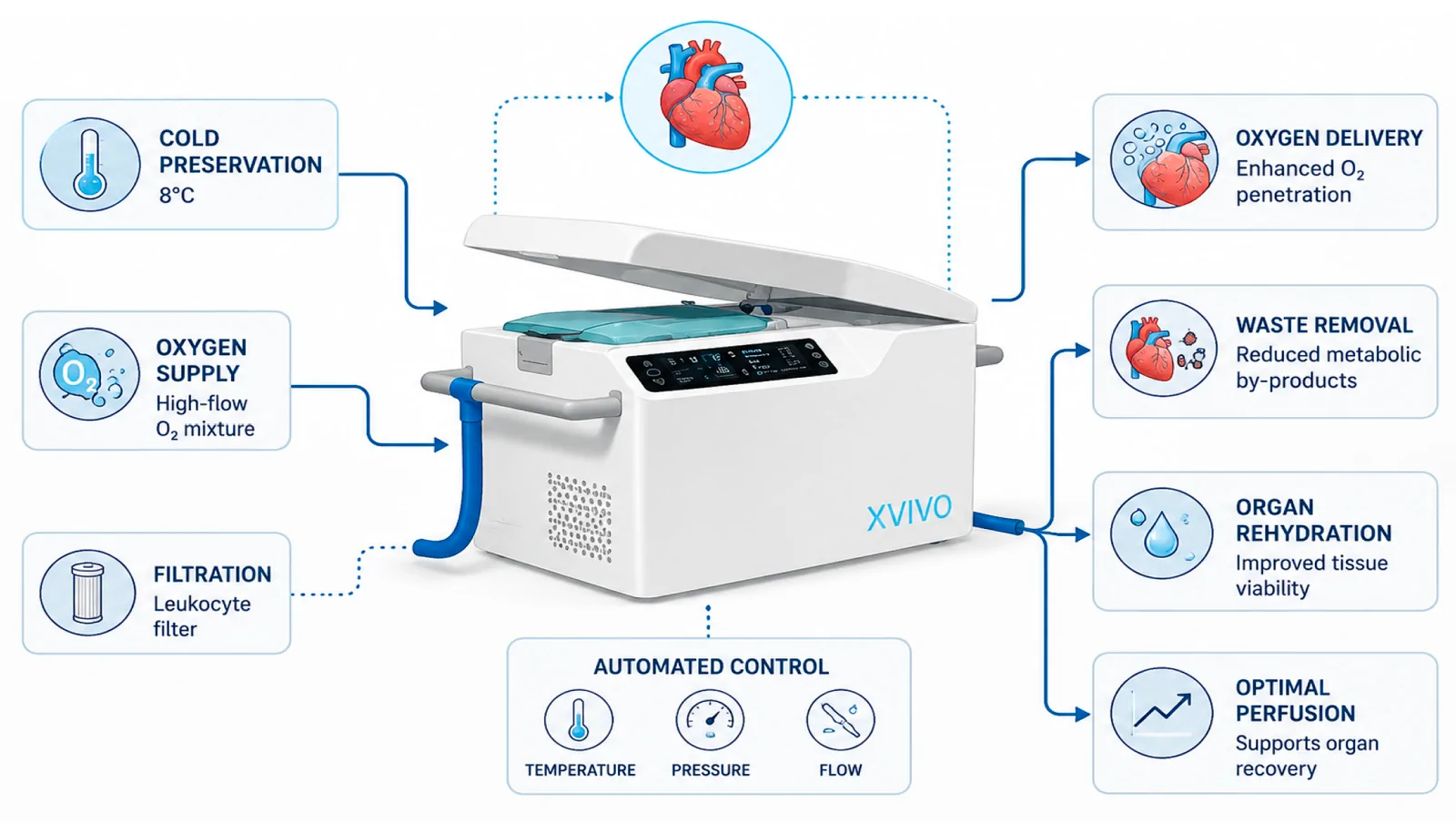
XVIVO HOPE system.

**Table 1:** Comparative overview of cardioplegic solutions used in heart transplantation: University of Wisconsin (UW), Celsior, and Histidine–Tryptophan–Ketoglutarate (HTK).

Feature	University of Wisconsin (UW)	Celsior	Histidine–Tryptophan– Ketoglutarate (HTK)
Typical arrest strategy	Often preceded by cardioplegic flush (CC + UW)	Single infusion; rapid distribution	Single-dose protocol; large volume
Reported clinical outcomes	Best early survival (30–90 days) and lower histologic necrosis [2,3]	Comparable to HTK; slightly inferior to UW for early outcomes [2]	Comparable to Celsior; inferior to UW for long-term survival [3]
Advantages	Excellent metabolic protection, proven efficacy	Low viscosity, easy handling	Simple, inexpensive, single-dose administration
Limitations	High viscosity, less ideal for initial flush; reduced efficacy >6 h CIT	Possibly higher endothelial activation, less protection with long CIT	Limited buffering and antioxidant capacity, suboptimal for marginal donors
Optimal application context	Standard and prolonged ischemia, marginal donor hearts when combined with CC + UW	Centres preferring low viscosity and rapid flush	Multi-organ retrieval, short CIT scenarios

Abbreviations: CC, custodiol cardioplegia; CIT, cold ischemia time; HTK, histidine–tryptophan–ketoglutarate; UW, University of Wisconsin. Numbers in square brackets refer to citations in the main text.

**Table 2:** Main clinical studies on donor heart preservation strategies.

Study	Year	Study Design	Preservation Strategy	Clinical Setting	Main Findings Relevant to This Review
Urban et al. [4]	2023	Clinical study	Paragonix SherpaPak Cardiac Transport System in DCD hearts	DCD heart transplantation	Reported favorable functional outcomes after transport of DCD donor hearts, supporting the feasibility of controlled hypothermic transport in this setting.
Bitargil et al. [6]	2022	Single-center comparative clinical study	SherpaPak vs. conventional ice cold storage	Adult heart transplantation	SherpaPak maintained controlled temperatures during transport and was associated with shorter ICU stay despite longer ischemic times; no severe PGD or mortality was observed in the SherpaPak group.
Lechiancole et al. [7]	2025	Comparative clinical study	Controlled hypothermia vs. ice-cold storage	High-risk heart transplantation using marginal grafts	Controlled hypothermia showed a trend toward lower moderate-to-severe graft dysfunction and fewer persistent bradyarrhythmias, with less severe histologic injury.
Alam et al. [8]	2023	Observational clinical study	Precision-controlled cardiac transport system vs. ice-cooled transport	Heart transplant recipients	Demonstrated early differences in donor-derived cell-free DNA levels, suggesting reduced graft injury with controlled transport.
Kaliyev et al. [9]	2019	Comparative clinical study	Custodiol vs. warm blood cardioplegia during transport with OCS	Donor heart transport with Organ Care System	Supported the role of OCS-based preservation in reducing ischemic injury and facilitating graft conditioning during transport.
Zhu et al. [11]	2023	Clinical outcomes study	Temperature-controlled hypothermic storage system	Heart transplantation	Reported favorable transplant outcomes using temperature-controlled hypothermic storage, reinforcing the clinical value of strict thermal control.
Moayedifar et al. [20]	2024	Multicenter registry analysis	Advanced preservation in extended-criteria donor hearts	Extended-criteria donor heart transplantation	Showed lower post-transplant mechanical circulatory support use and lower severe PGD despite longer transport distances and ischemic times, with similar 1-year survival.
Wang et al. [13]	2025	Translational study with clinical/registry analysis	Crystalloid cardioplegia plus UW vs. standard UW preservation	Donor heart preservation with prolonged ischemia	Suggested that an initial crystalloid cardioplegic flush combined with UW solution improves tolerance to prolonged cold ischemia and may improve long-term survival.
Kaliyev et al. [12]	2020	Comparative clinical study	Normothermic ex vivo preservation with OCS vs. cold storage	Heart transplant recipients bridged with ventricular assist devices	OCS significantly reduced total ischemic time and reduced the need for post-transplant mechanical circulatory support.
Shudo et al. [15]	2023	Multicenter registry/comparative analysis	SherpaPak vs. conventional ice storage	Adult heart transplantation	Controlled hypothermic transport was associated with significantly lower severe PGD, including in propensity-matched analysis and in grafts with ischemic times >4 h.
Farhoud et al. [16]	2025	Matched comparative study	SherpaPak with prolonged ischemic time vs. conventional ice storage with short ischemic time	Heart transplantation	Despite markedly longer transport distance and ischemic time, SherpaPak preservation achieved similar 30-day survival and was associated with lower inotrope requirements and less right ventricular dysfunction.
Stehlik et al. [17]	2025	Clinical registry study	Normothermic pulsatile organ perfusion (OCS registry)	Heart transplantation	Provided contemporary registry-based evidence supporting the clinical utility of normothermic perfusion, particularly in higher-risk and expanded-criteria settings.
Rega et al. [18]	2024	Randomized controlled multicenter open-label trial	Hypothermic oxygenated perfusion (HOPE) vs. static cold storage	Donor heart transplantation	HOPE reduced primary graft dysfunction, severe PGD, and major adverse cardiac transplant events within 30 days compared with conventional static cold storage.

## Data Availability

No new data were created or analyzed in this study. Data sharing is not applicable to this article.

## Abbreviations

The following abbreviations are used in this manuscript:

DCD	Donation-after-circulatory-death
SCS	Static cold storage
UW	University of Wisconsin (solution)
HTK	Histidine–tryptophan–ketoglutarate
PGD	Primary graft dysfunction
SCTS	SherpaPak Cardiac Transport System
OCS	Organ Care System
CIT	Cold ischaemic time
HR	Hazard ratio
CI	Confidence interval
RD	Risk difference
CC	Crystalloid cardioplegia
ET	Endothelin
iNOS	Inducible nitric oxide synthase
ECD	Extended-criteria donor
ICU	Intensive care unit
MCS	Mechanical circulatory support
OR	Odds ratio
RCT	Randomized controlled trial
HOPE	Hypothermic oxygenated perfusion
